# Development and validation of image quality scoring criteria (IQSC) for pediatric CT: a preliminary study

**DOI:** 10.1186/s13244-019-0769-8

**Published:** 2019-09-23

**Authors:** Atul M. Padole, Pallavi Sagar, Sjirk J. Westra, Ruth Lim, Katherine Nimkin, Mannudeep K. Kalra, Michael S. Gee, Madan M. Rehani

**Affiliations:** Department of Radiology, Massachusetts General Hospital, Harvard Medical School, 75 Cambridge Street, Suite 244, Boston, MA 02114 USA

**Keywords:** Pediatric CT, Clinical indications, Image quality scoring criteria, Radiation protection, Radiation dose optimization

## Abstract

**Objective:**

To develop and assess the value and limitations of an image quality scoring criteria (IQSC) for pediatric CT exams.

**Methods:**

IQSC was developed for subjective assessment of image quality using the scoring scale from 0 to 4, with 0 indicating desired anatomy or features not seen, 3 for adequate image quality, and 4 depicting higher than needed image quality. Pediatric CT examinations from 30 separate patients were selected, five each for routine chest, routine abdomen, kidney stone, appendicitis, craniosynostosis, and ventriculoperitoneal (VP) shunt. Five board-certified pediatric radiologists independently performed image quality evaluation using the proposed IQSC. The kappa statistics were used to assess the interobserver variability.

**Results:**

All five radiologists gave a score of 3 to two-third (67%) of all CT exams, followed by a score of 4 for 29% of CT exams, and 2 for 4% exams**.** The median image quality scores for all exams were 3 and the interobserver agreement among five readers (acceptable image quality [scores 3 or 4] vs sub-optimal image quality ([scores 1 and 2]) was moderate to very good (kappa 0.4–1). For all five radiologists, the lesion detection was adequate for all CT exams.

**Conclusions:**

The image quality scoring criteria covering routine and some clinical indication-based imaging scenarios for pediatric CT examinations has potential to offer a simple and practical tool for assessing image quality with a reasonable degree of interobserver agreement. A more extensive and multi-centric study is recommended to establish wider usefulness of these criteria.

**Electronic supplementary material:**

The online version of this article (10.1186/s13244-019-0769-8) contains supplementary material, which is available to authorized users.

## Key points

- There are limitations of both the objective and subjective assessments of image quality
- The study developed an image quality scoring criteria (IQSC) to help in subjective assessment of pediatric CT
- The interobserver agreement among five radiologists for acceptable and non-acceptable image quality was moderate to very good
- The IQRS offers a simple and practical tool for assessing image quality in pediatric CT exams with a reasonable degree of interobserver agreement

## Introduction

Computed tomography (CT) plays a vital role in pediatric patients. For any action on making CT safer for children, the principle of optimization stipulated by the International Commission on Radiological Protection (ICRP) requires balancing radiation dose with the image quality so that necessary diagnostic information is not compromised [1, 2]. ICRP has recommended that image quality in CT should be not higher than needed for confident diagnosis [3]. Pediatric CT protocols must be tailored based on clinical indications to ensure that CT radiation dose to pediatric patients is appropriate [4–8]. Several studies during the last half of a century have tried to assess image quality objectively [9–13]. Parameters like quantitative image noise, signal-to-noise ratio, contrast-to-noise ratio, modulation transfer function (MTF), normalized noise power spectrum (NNPS), detective quantum efficiency (DQE), contrast details, and forced-choice just noticeable difference (JND) have been studied. Most objective scoring criteria are useful for academic studies rather than for applying in day-to-day practice in clinical situations globally. The results show that a particular image quality parameter is sensitive in detecting a specific aspect of information in the image but inadequate as an overall measure of image quality in terms of clinical usefulness. Subjective scoring systems for image quality and some using image noise, contrast, sharpness, and artifacts have been applied to CT image quality [13–16]. Such studies focus on the visual aspect of quality. The subjective judgment of images by a radiologist has been used routinely in day-to-day life, and it does provide a powerful tool to judge many aspects of image quality and information content simultaneously as needed for diagnosis. Some papers have compared the subjective scoring of images with objective indices and have confirmed the usefulness of subjective scoring [9, 10]. Generally, individual preferences of images’ quality acceptability lead to variability in image quality and radiation dose. The interobserver variability is considered a substantial source of the problem that leads to inconsistent and inadequate subjective image quality assessments. If the criteria developed are such that they have a lower interobserver variability, it may become widely acceptable.

A recent publication has introduced a new concept of acceptable quality dose (AQD) and emphasized the need for developing criteria for satisfactory image quality to integrate subjective image quality aspects with radiation dose for optimization studies [17]. A method of image quality criteria that can be widely accepted in the context of non-academic centers and have less interobserver variability is needed [18]. Such an approach would assist in CT radiation dose optimization as well as dose survey studies of image quality [17]. Combining complex issues of service delivery into a single score of assessment like the option of a five-star rating system in most service providers provides an easy and uncomplicated way to assess user satisfaction.

In the backdrop of the above scenario, we developed the image quality scoring criteria for CT images of children with the following objectives

- To reflect upon adequate visualization of pertinent anatomical structures (kidney, appendix, skull) based on specific clinical indication (kidney stone, appendicitis, craniosynostosis) with appropriate radiation dose
- To score the image for its usefulness for the purpose and thus be clinical indication based
- To have criteria that are practicable in busy clinical practice
- To introduce subjective image quality in dose and image quality optimization studies
- To achieve practicability in less-resourced countries without measurement parameters and with the subjective judgment of the radiologists

For this study, we propose image quality scoring criteria (IQSC) for the assessment of pediatric CT image quality for routine and clinical indication-based situations. Further, the goal of our study was to assess the diagnostic acceptability and interobserver variability of pediatric CT with this clinical indication-based image quality scoring criteria (IQSC).

## Materials and methods

This study was performed as a quality improvement project and thus exempted from the need for approval from the institutional review board (IRB). MKK received research grants from Siemens Healthineers and Riverian Inc. for unrelated projects. Other co-authors have no pertinent financial disclosure.

### Selection of CT images of pediatric patients

The CT protocols in this study were selected based on their frequent use in most pediatric practices and not necessarily to assess for low-contrast detectability of lesions.

For this study, pediatric CT exams were searched and randomly selected from Render, a web-based radiology search engine and database of Massachusetts General Hospital (MGH). Routine and clinical indication-based pediatric CT exams from 30 separate pediatric patients were selected, five each for routine chest (*n* = 5, mean age 8 ± 4 years), routine abdomen (*n* = 5, 12 ± 5 years), kidney stone (*n* = 5, 16 ± 1 years), appendicitis (*n* = 5, 10 ± 3 years), craniosynostosis (*n* = 5, 3 ± 5 years), and ventriculoperitoneal (VP) shunt (*n* = 5, 6 ± 5 years). The patient selection was done regardless of pediatric age, gender, weight, and CT radiation dose (CTDIvol and DLP). CT exams were saved, and a work list was created on picture archiving and communication system (PACS, AGFA Impax 6.3, AGFA Healthcare, Belgium) for image quality evaluation using proposed IQSC (detailed description in the Additional file 1). Radiologists were allowed to view the entire CT examination including relevant multiplanar images (sagittal and coronal planes for chest and abdomen CT) and any volume rendered images (for craniosynostosis). The section thickness for head and chest CT images was 2.5 mm and 5 mm for abdomen CT images. Patients’ demographics, weight, height, and CT radiation dose information (CTDIvol, DLP) were recorded. For each pediatric patient, body mass index (BMI, kg/m^2^) was calculated from their weight and height.

### Subjective image quality evaluation

In consideration of all the points identified in the introduction above, the scoring scale was decided from 0 to 4, with 0 indicating desired features not seen, 3 for adequate image quality, and 4 depicting higher than needed quality (Table 1 and Additional file 1). Although the score of 3 or 4 both suggest acceptable image quality for clinical purpose, the score of 4 represents higher than necessary radiation dose and need for protocol optimization. The details of IQSC are included in Additional file 1. Five board-certified pediatric subspecialty radiologists (experience ranging from 5 to 20 years) participated in an independent evaluation of selected CT examinations. Selected pediatric CT exams were displayed for the scoring of subjective image quality on PACS (AGFA Impax 6.3, AGFA Healthcare, Belgium) under similar ambient conditions as used for the standard of care radiology reporting. Radiologists could change the window width and levels as per their preferences. The clinically important lesions were recorded in addition to lesions size, number, location, and attenuation. Lesion conspicuity and visibility of anatomical structures were assessed using an IQSC described in Table 1 and Additional file 1. Although a number of anatomic structures were mentally assessed, each radiologist gave a single global IQSC score for each exam.

**Table 1:** Scoring chart for image quality scoring criteria (IQSC)

0s = Desired features not seen	
0i = Anatomy not included in the images	
1 = Unacceptable quality (images do not allow diagnostic interpretation)	
2 = Limited quality (images are adequate only for limited clinical interpretation due to high noise*)	
3 = Adequate quality (images are just adequate for diagnostic interpretation)	
4 = Higher than needed quality (images are much better than needed for interpretation: images with little or no noise)	

*Noise is described as salt and pepper appearance of the image

### Statistical analysis

Statistical software (SPSS 21, IBM, Armonk, NY) was used to analyze the data. The average, median, and frequency of subjective IQSC score for all six clinical indications were calculated. The median subjective IQSC were also included. Student’s *t* test was used to evaluate the subjective image quality. The *p* value of 0.5 with 95% confidence interval was considered significant. The kappa statistics were used to assess the inter-observer variability of image quality (scores 3 or 4) vs suboptimal image quality (scores 1 and 2) based on the kappa values (poor < 0.2, fair 0.2–0.4, moderate 0.4–0.6, good 0.6–0.8, very good 0.8–1).

## Results

Table 2 summarizes mean ± standard deviation (SD) age, gender, weight, and BMI, CTDIvol and DLP for different CT protocols of included patients. The median subjective image quality scores based on IQSC are summarized in Table 3. Frequency distribution of IQSC for routine and various clinical indication based CT protocols is summarized in Table 4.

**Table 2:** Mean ± standard deviation of age, gender, weight, and body mass index (BMI), CTDI_vol_, and DLP of patients included in the study

	Age (years)	Male to female	Weight (kg)	BMI (kg/m^2^)	CTDIvol (mGy)	DLP (mGy cm)
Routine chest	8 ± 4	0:5	32 ± 17	18 ± 3	1.97 ± 0.7	63 ± 18
Routine abdomen	12 ± 5	1:4	39 ± 19	18 ± 4	6 ± 4.3	258 ± 227
Kidney stone	16 ± 1	1:4	64 ± 16	24.5 ± 5	3.7 ± 1.7	149 ± 83
Appendicitis	10 ± 3	3:2	38 ± 13	18.4 ± 3	6 ± 2.5	207 ± 80
Craniosynostosis	3 ± 4.5	3:2	16 ± 15	17.6 ± 2	1.4 ± 0.1	25 ± 2
VP shunt	6 ± 5	1:4	26 ± 18	18 ± 5	19 ± 13	337 ± 240

**Table 3:** Median IQSC for different routine and clinical indication-based CT protocols

	Reader 1	Reader 2	Reader 3	Reader 4	Reader 5	Median
Routine chest	3	3	3	3	4	3
Routine abdomen	3	4	3	3	4	3
Kidney stone	3	4	3	3	4	3
Appendicitis	4	4	3	3	3	3
Craniosynostosis	3	3	3	3	2	3
VP shunt patency	3	4	3	3	3	3
Median	3	4	3	3	4	3

**Table 4:** Frequency of subjective image quality score (1–4) for the five radiologists

	Score 1 (%)	Score 2 (%)	Score 3 (%)	Score 4 (%)
Routine chest CT	–	–	16 (64%)	9 (36%)
Routine abdomen CT	–	–	15 (60%)	10 (40%)
Renal stone CT	–	1 (4%)	18 (72%)	6 (24%)
Appendicitis CT	–	2 (8%)	16 (64%)	7 (28%)
Craniosynostosis CT	–	3 (12%)	18 (72%)	4 (16%)
VP shunt CT	–	–	18 (72%)	7 (28%)
Total (%)	–	6 (4%)	101 (67%)	43 (29%)

### Subjective image quality for routine chest CT

**Fig. 1:**
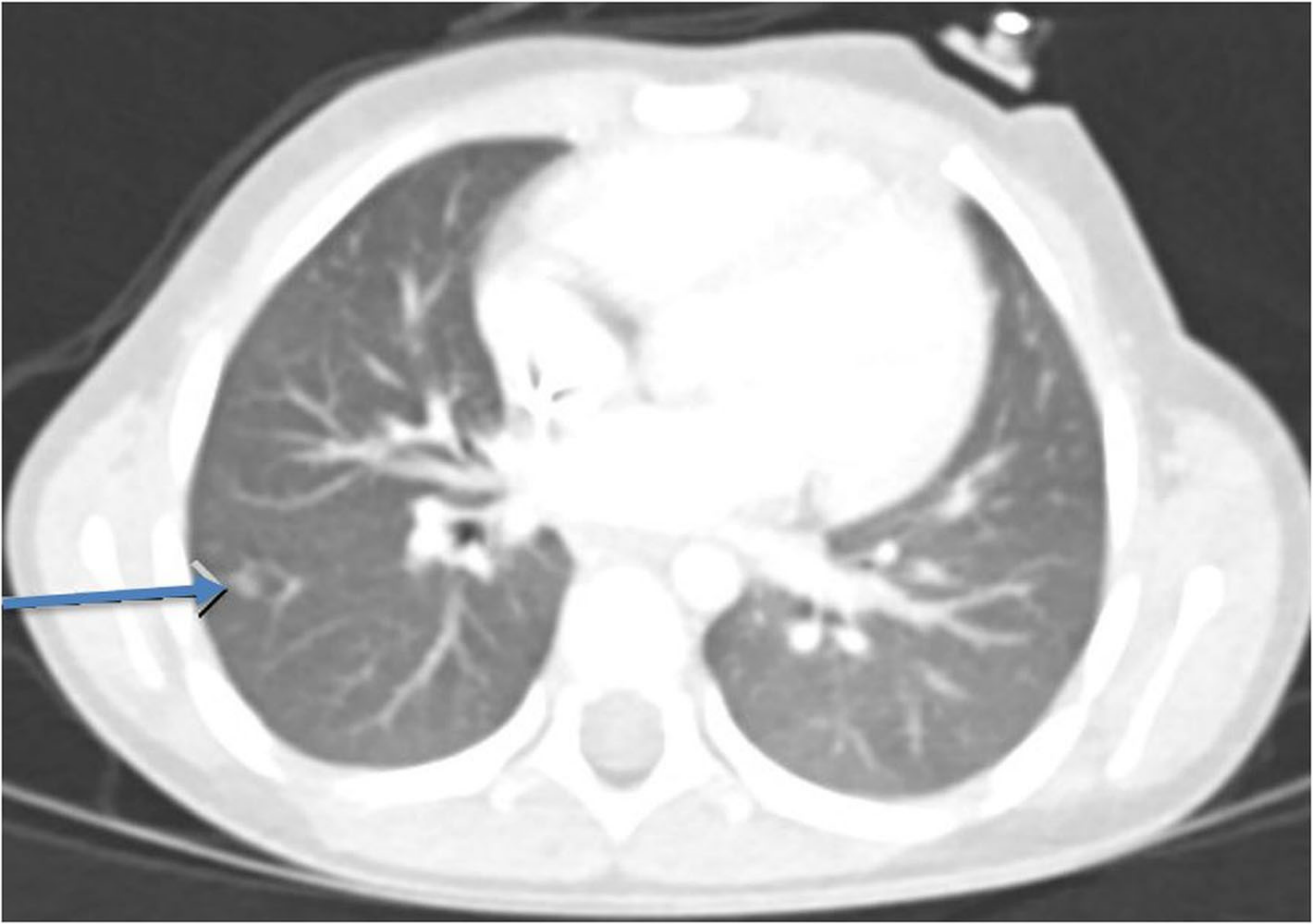
Transverse chest CT image of a 4-year-old girl (15 kg, CTDIvol 1.4 mGy). There is a subcentimeter nodule in the right lower lobe (arrow). Median IQSC score was 3

There were a total of nine lesions detected on CT which included ground-glass opacities (*n* = 4), pulmonary nodule (*n* = 1, Fig. 1), enlarged right hilar nodes (*n* = 1), enlarged subcarinal lymph node (*n* = 1), paratracheal lymph nodes (*n* = 1), and hepatic steatosis (*n* = 1). Lesion detection was unaffected, and no false-positive lesions were detected on these routine chest CT exams. For all routine chest CT exams, the subjective image quality was acceptable for diagnostic interpretation (score 3 or score 4) for readers. All CT exams were either score 3 or 4 by all five radiologists. The median image quality scores for routine chest CT exams for the readers were 3 (Table 3).

Although there were variations in subjective image quality scores, all radiologists agreed on studies deemed diagnostically useful (scores of 3 [16/25] and 4 [9/25] as in Table 4). For routine chest CT, the interobserver agreement among five readers (acceptable image quality [scores 3 or 4] vs suboptimal image quality [scores 1 and 2]) was very good (*p* < 0.0001). For all the five readers, the frequency of subjective image quality score was 64% (score 3) and 36% (score 4) for routine chest CT exams (Table 4).

### Subjective image quality for routine abdomen CT

**Fig. 2:**
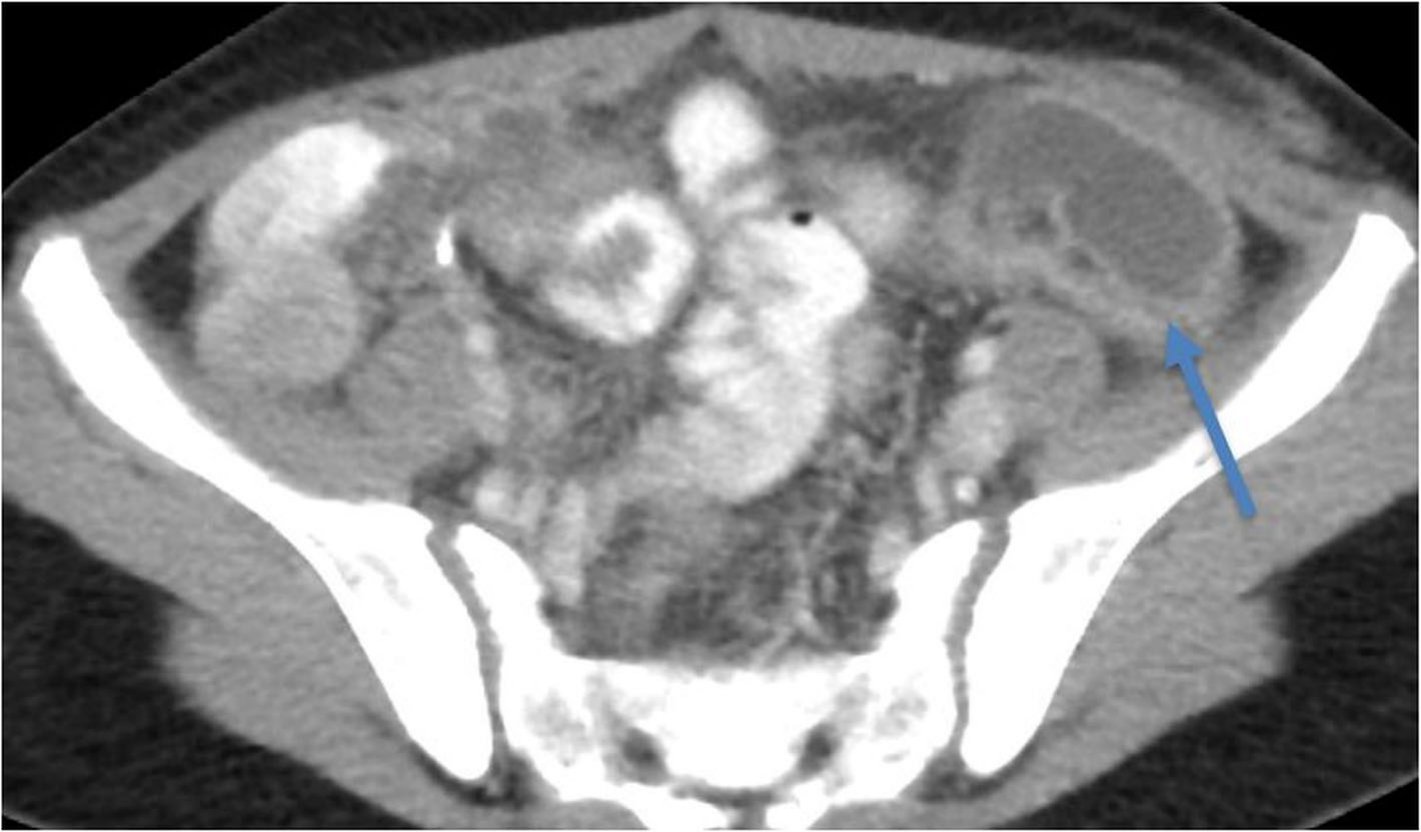
Transverse abdomen CT image of a 14 yrs. M (42 kg) acquired at 4.4 mGy. Interloop abscess (arrow) and overall image quality scored optimal (scores 3 or 4) by all readers

The nine detected lesions on routine abdominal CT included intra-abdominal fluid collections or interloop abscesses (*n* = 3, Fig. 2), enlarged appendix (*n* = 1), mesenteric lymphadenopathy (*n* = 2), acute appendicitis (*n* = 1), colonic diverticulosis (2), and trace ascites (*n* = 1). The image quality scores for routine abdomen/pelvis CT exams for all five readers were 3 or 4. No CT exam was scored 1 or 2 by all the five radiologists. The median image quality scores for routine abdomen CT exams for readers were 3 (Table 3). The percentage of frequency of subjective image quality score for the five readers was 60% (score 3) and 40% (score 4) (Table 4). The interobserver agreement among five readers for acceptable vs sub-optimal image quality was very good (kappa = 1).

### Subjective image quality for kidney stone CT

**Fig. 3:**
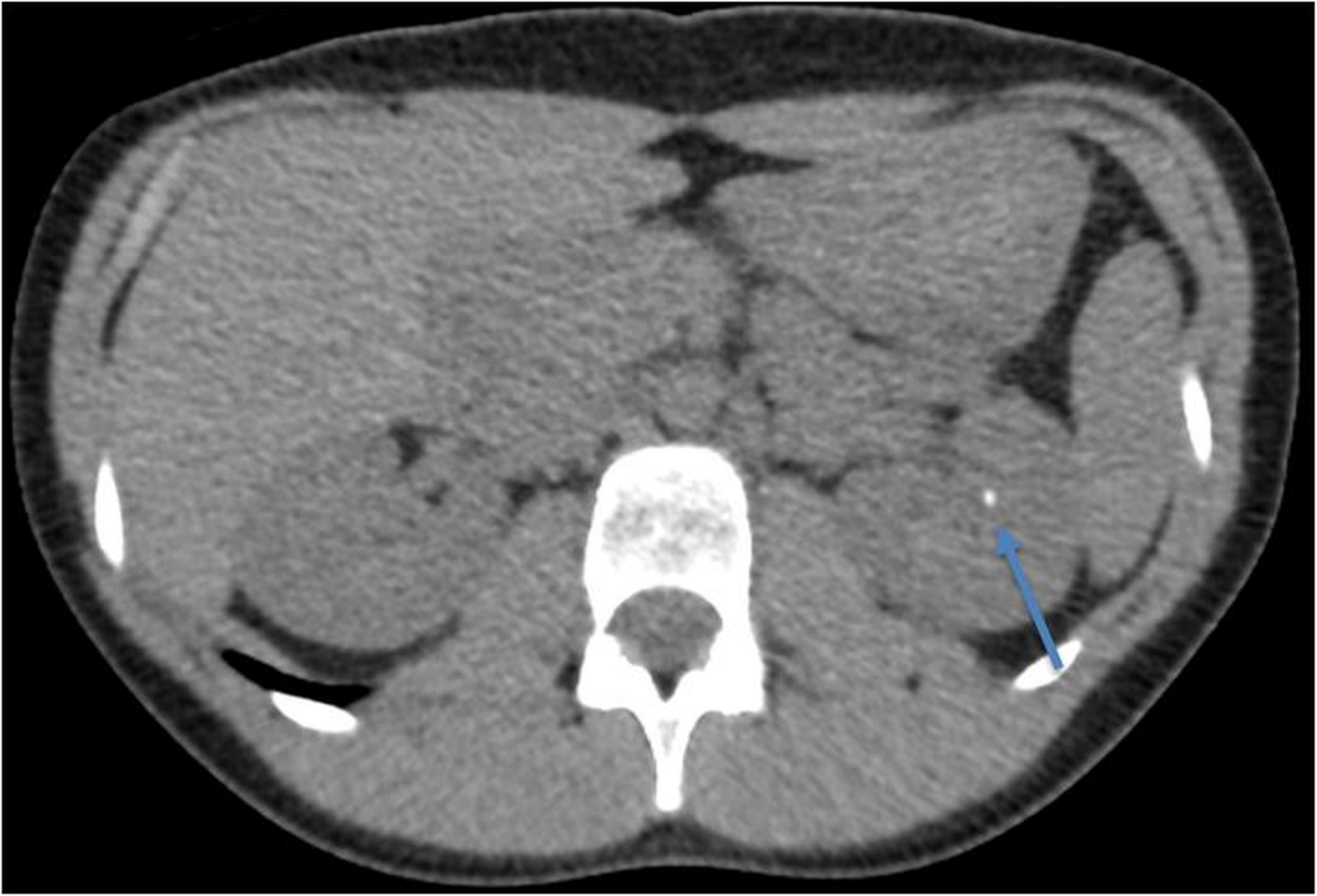
Transverse abdomen CT image (kidney stone protocol) of a 17-year-old female (53 kg) acquired at 2.3 mGy. Left kidney stone (arrow) and overall image quality scored optimal by four-fifths of the readers and sub-optimal by one-fifths of the readers. However, a diagnosis of kidney stone was unaffected by all readers. Median IQSC score was 3

There were 13 kidney stones (3–5 mm, Fig. 3) and a case of hydronephrosis detected on kidney stone CT exams. The renal stones detection was unaffected on all five CT exams for all readers. The median image quality scores for kidney stone CT exams for the readers were 3 (Table 3). The interobserver agreement between five readers was very good (0.8–1). The percentage of frequency of subjective image quality score for the five readers were 4% (score 2), 72% (score 3), and 24% (score 4) (Table 4).

### Subjective image quality for appendicitis CT

**Fig. 4:**
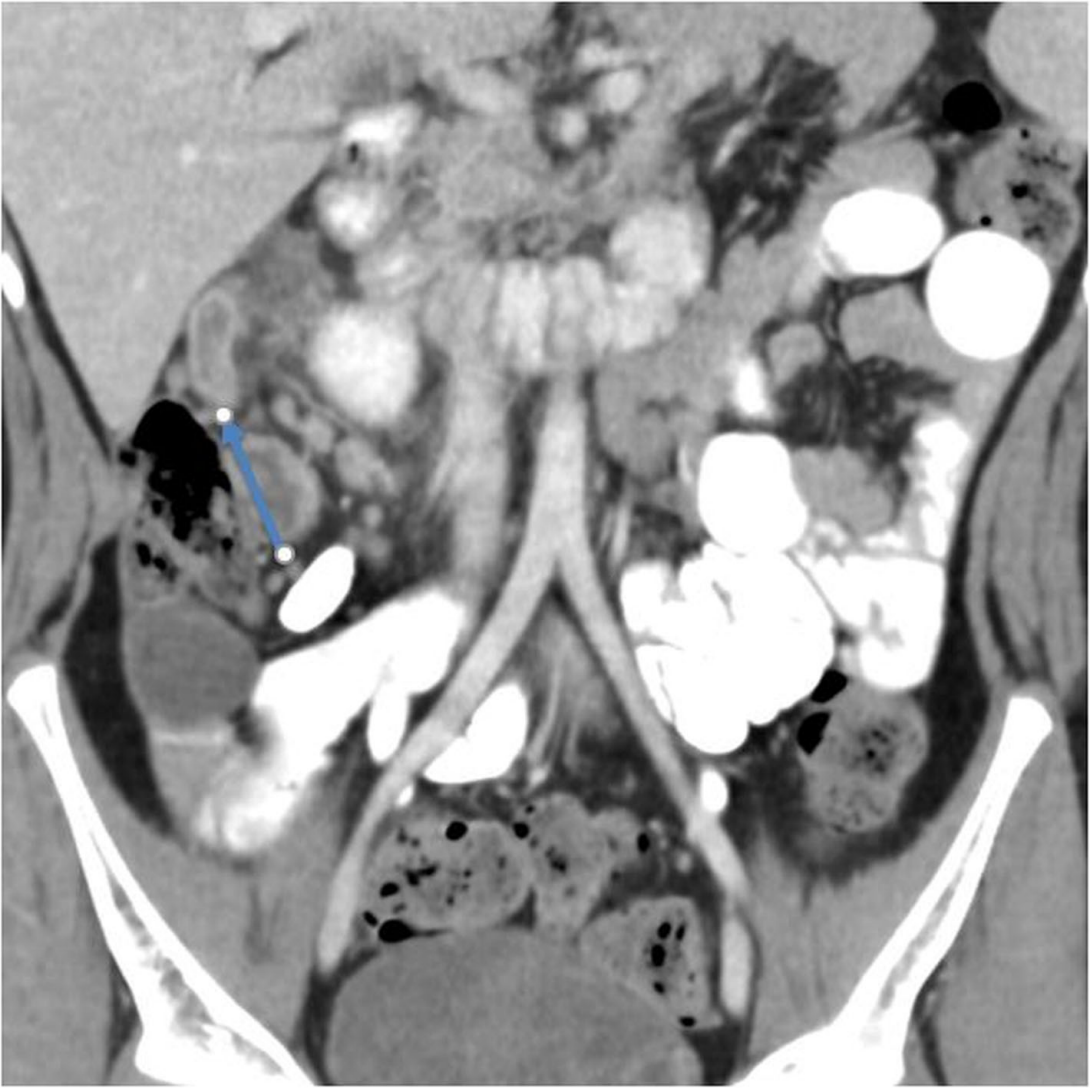
Coronal abdomen CT image (appendicitis protocol) of an 11-year-old male (42 kg) acquired at 6 mGy. Acute appendicitis (arrow) and overall image quality scored optimal (score 3 or 4) by all readers

The seven lesions detected on appendicitis CT exams included acute complicated appendicitis (*n* = 3, Fig. 4), acute uncomplicated appendicitis (*n* = 2), splenic hypodensity (*n* = 1), and mesenteric adenitis (*n* = 1). The median image quality scores for appendicitis CT exams for readers were 3 (Table 3). The interobserver agreement between five readers was good to very good (0.6–1). The subjective image quality scores for the five readers were 8% (score 2), 64% (score 3), and 28% (score 4) (Table 4).

### Subjective image quality for craniosynostosis CT

**Fig. 5:**
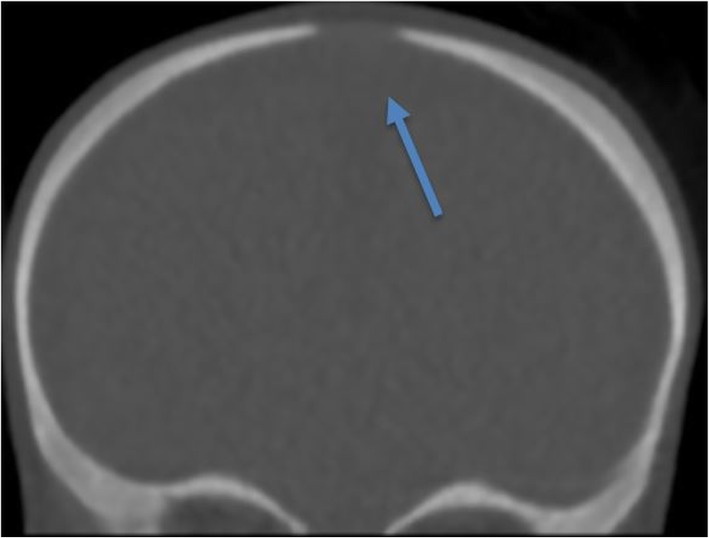
Coronal head CT image (craniosynostosis protocol) of a 4-month-old male (7 kg) acquired at 1.5 mGy. Anterior fontanelle without evidence of craniosynostosis (arrow) and overall image quality scored optimal by all readers

The findings on craniosynostosis CT exams included premature closure of the metopic suture anatomically, posterior fossa malformation, anterior fontanelle without evidence of craniosynostosis (Fig. 5), right hemicraniectomy, and cranioplasty. For one-fifth of the readers, the three sub-optimal or limited craniosynostosis CT exams had motion artifacts affecting the diagnostic interpretation. The median image quality scores for craniosynostosis CT exams for the readers were 3 (Table 3). The interobserver agreement for subjective image quality among five readers was moderate to very good (0.4–1). For the five readers, the percentage of frequency of subjective image quality score was 12% (score 2), 72% (score 3), and 16% (score 4) (Table 4).

### Subjective image quality for VP shunt CT

**Fig. 6:**
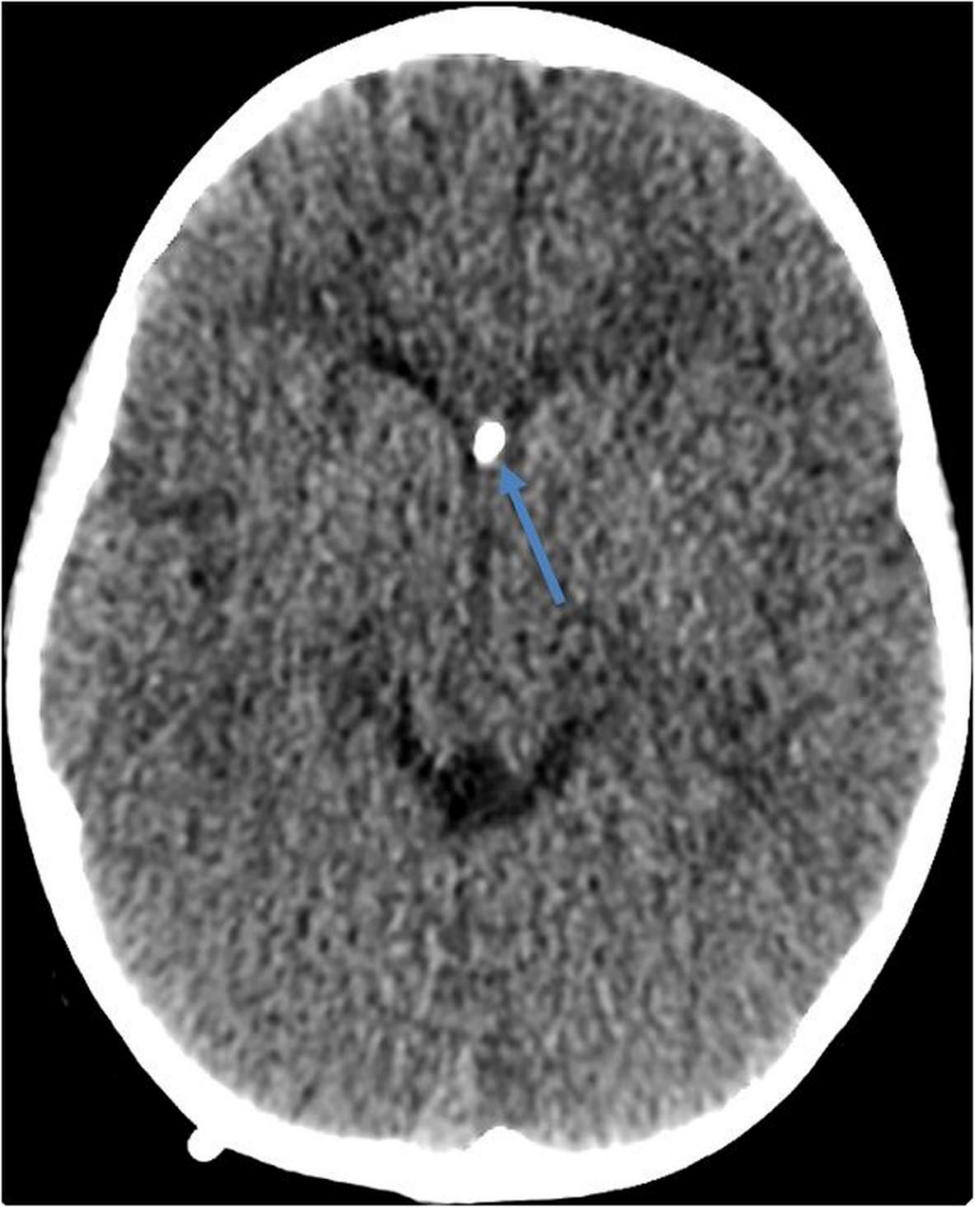
Transverse head CT image (VP shunt patency protocol) of a 7-year-old female (20 kg) acquired at 6 mGy. Hydrocephalus with VP shunt (arrow) and overall image quality scored optimal by all readers

The findings on VP shunt CT exams include severe hydrocephalus with VP shunt (Fig. 6), cyst in the left maxillary, sphenoid sinus inflammation, burr hole, left occipital shunt catheter, encephalomalacia, cranioplasty flap, and periventricular white matter hypodensities. The subjective image quality scores for VP shunt CT exams for four-fifths of the readers were 3 or 4 and for one-fifth of the readers, the score was 3. Although one-fifth of the readers gave suboptimal scores for two VP shunt CT exams, these were unrelated to the acquisition parameters and were related to intraventricular hemorrhage and implanted VP shunt port in one patient and motion artifacts affecting the diagnostic interpretation in other patients. Following adjudication, scores for both exams were revised to three following adjudication. The median image quality scores for VP shunt CT exams for readers were 3 (Table 3). The interobserver agreement for subjective image quality among five readers was very good (0.8). For the five readers, the percentage of frequency of subjective image quality score was 72% (score 3), and 28% (score 4) (Table 4).

## Discussion

The reasonable degree of agreement in this preliminary study among different observers is encouraging for further work in this area and has the promise of a simple tool for image quality scoring that can be made practicable.

In the 1990s, there were efforts in developing image quality criteria notably through the work of European Commission projects [15, 16]. Initially, quality criteria were developed for radiographic images including mammography, and later, also for CT [15, 16, 19]. These criteria provide an educational tool so that users could become aware of the essential features in the radiological image. However, despite two decades of existence, there is paucity of information on these criteria in dose survey studies and in achieving optimization of dose and image quality in clinical practice. Most publications focus on image quality criteria based on body regions rather than specific clinical indications in each body region [13, 14, 20–25]. Our study simplified image quality assessment based on subjective judgments rather than measurable values while making it more specific and relevant to clinical indications.

The driving force for the development of the scoring system was the broader global feasibility and applicability, relying upon subjective assessment of imaging specialist rather than downplaying it. Unfortunately, there is a general tendency of rejecting the variability in human perception, whereas the same is not the case with machines. The radiation dose for the same radiological examination varies widely even using the same type of equipment from the same manufacturer and with patients of similar body size [25] (see Table 4). Variability is part of research studies. We need to assess the variability and find ways to reduce it rather than getting deterred by it. The fact that there is substantial variability among interpreters of images is well known. We evaluated variability in image quality scoring.

The IQSC is an attempt to fill the current gap of a need to integrate image quality in terms of information content with radiation dose. Adequate visualization of relevant structures in the image formed the basis for scoring to depict information content for specific clinical indications. For example, in the case of craniostenosis CT examinations, sutures and skull shape are important while brain parenchyma is not. On the other hand, for VP shunt evaluation CT, demonstration of ventricular systems is the key. In a study of children with suspicion of craniosynostosis, the image quality was assessed using parameters’ image noise, image sharpness, overall diagnostic acceptability, and artifacts [26]. In another study, a subjective three-point scale ranging from very good to non-diagnostic image quality rating of perfusion disturbance, intracranial peripheral vessel depiction, and motion or streak artifacts was used [27].

**Fig. 7:**
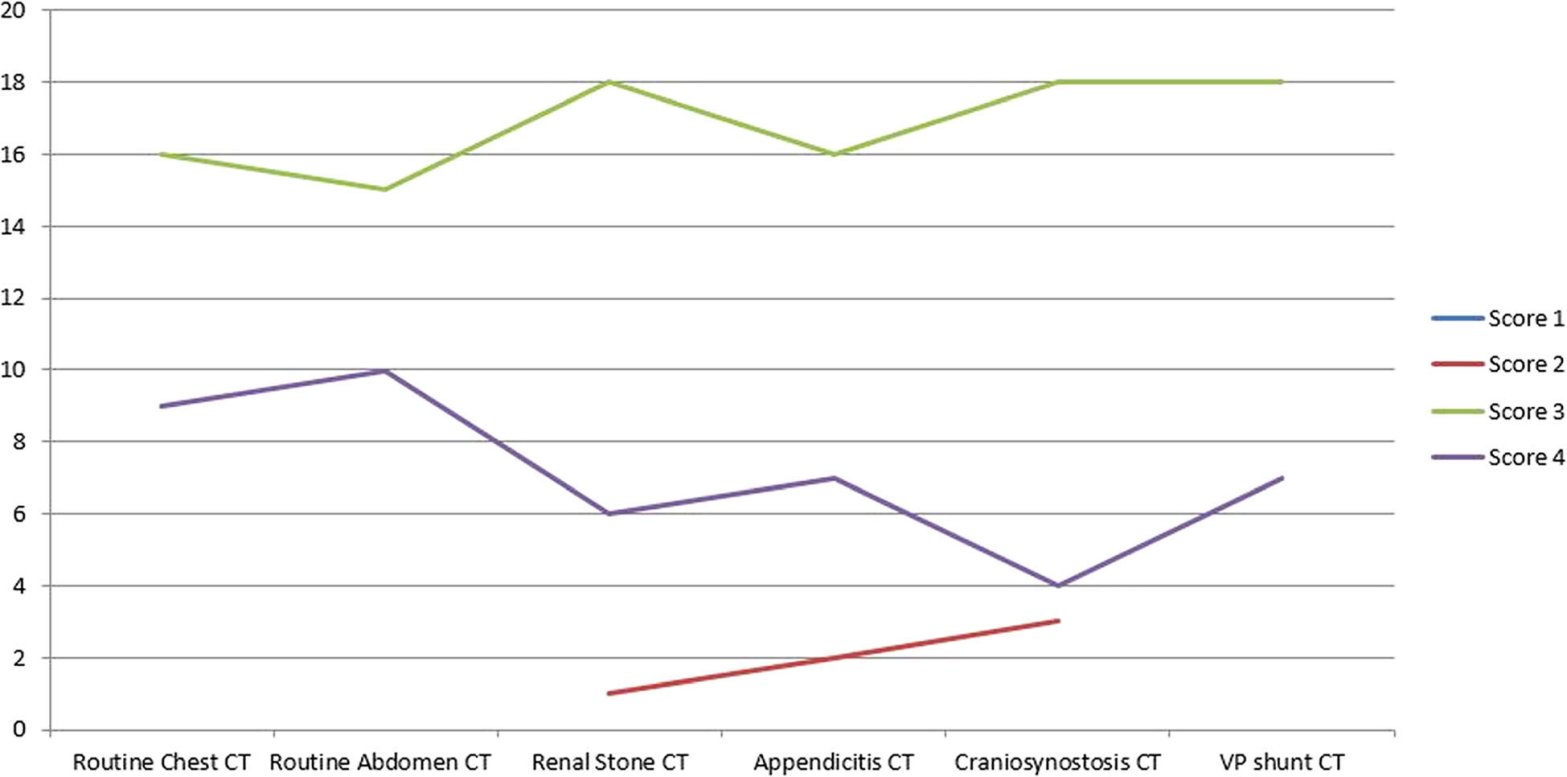
Frequency graph of subjective image quality score (1–4) for five study readers

In our study, for all five radiologists, the lesion detection was unaffected for all CT exams. For five readers and when we take the full picture of all examinations combined, two-third (67%) assessments were leading to a score of 3, 29% to score of 4, and only 4% to score of 2 (Table 4, Fig. 7). When scores 3 or 4 are combined to represent acceptable image quality scores, they cover 96% of situations. The median image quality scores for all pediatric CT exams for readers were the score of 3. For all pediatric CT exams, the interobserver agreement among five readers (acceptable image quality [scores 3 or 4] vs sub-optimal image quality ([scores 1 and 2]) was moderate to very good (kappa 0.4–1). The exception for the score of 2 (in 4%) cases was seen due to the presence of motion artifacts and non-inclusion of the anatomy in one case. The same radiologist also rated three cases of craniostenosis CT as suboptimal or limited due to the inability of assessing third and fourth ventricles, which do not represent the target regions with this protocol. Therefore, the radiation doses with VP shunt patency CT (for assessment of ventricular system) are higher than for the craniostenosis CT protocol.

It must be pointed out that the radiologists were provided the scoring criteria but did not receive prior training based on the scoring system to avoid any effect on interobserver variation. The latter was important since we wanted to assess the baseline variability.

Differences between subjective image quality scores 3 or 4 indicate that some radiologists are more radiation dose conscious than others and this creates the need for training and culture development on acceptance of low dose CT images (score 3) which does not have the image appearance of higher dose images (score 4). It has been shown that training leads to acceptance of low dose CT images and radiation dose optimization [23]. This paper showed that post-training image quality assessed subjectively in terms of artifacts, gray-white matter differentiation, and visualization of posterior fossa structures, and the need for repeat CT examination was not inferior to pre-training levels for most items. Our study adds an element of quantification of image quality based on the described criteria.

The balance among CT image quality and radiation dose is important; hence, image quality scoring criteria needs to be simple and practical for image quality considerations in dose surveys. Therefore, we propose IQSC for better practicability and the improvement of subjective assessments. It must be emphasized that objective assessments have several limitations over subjective assessments in terms of a limited aspect of image quality that each objective parameter assesses. On the other hand, the subjective evaluation can simultaneously compare several features. Indeed, modern medicine is largely dominated by subjective assessments and individual judgments. Clinical interpretation of radiological images is predominantly subjective. While it is vital to make progress in objective models, it is equally important to keep on making advancement towards enhancing the utility of observer models or scoring criteria to fully utilize human potential [28–30]. We believe that IQSC can aid in radiation dose optimization in children.

The scoring system is not meant to look at which aspect contributed to the overall score. It is the same when the noise index is measured and used as a criterion for image quality. Noise index does not convey any idea about diagnostic value or diagnostic quality of the image. Our study focuses on overall image quality rather than defining specific characteristics.

## Limitations and clarity on limitation

The sample size of our study was small since this is an initial pilot baseline study. Our study assessed a limited number of CT protocols (*n* = 6), but nonetheless, it provides a template for evaluation of subjective image quality evaluation for other CT protocols. We did not exclude patients with motion artifacts from our study, which led to some interobserver variation on the issue other than the intended subject of radiation dose. The latter was intentional since we wanted to include real-life cases of CT in children where motion artifacts are common and at times unavoidable despite best attempts. In future studies with a larger sample, there is a need to establish a correlation of IQSC with dose. Technical problems with the scanner are outside the scope of this. Notwithstanding the impact of equipment and operational aspects like bolus timing, incomplete coverage, excessive patient motion, and imaging performed at expiration instead of inspiration, the purpose of a single value of image quality is and cannot be a representative of cause analysis of poor quality. The score provides the need for cause analysis.

Assessment of image quality in our study was limited to one institution and pediatric radiologists only. This calls for extensive and multi-centric study to establish the usefulness of these criteria.

## Conclusions

The image quality scoring criteria (IQSC) for routine and also clinical indication-based imaging scenarios for pediatric CT protocols provide a simple and practical tool for assessing image quality with a reasonable degree of interobserver agreement. The score can trigger the need for cause analysis. The study calls for an extensive and multi-centric study to establish the usefulness of these criteria.

## Additional file

Additional file 1: Image quality scoring criteria (IQSC) for pediatric CT. (DOCX 23 kb)

## Data Availability

All data generated or analyzed during this study are included in this published article (and its supplementary information files). Further data, if needed, is available from the corresponding author.

## Abbreviations

AQD: Acceptable quality dose
CT: Computed tomography
DQE: Detective quantum efficiency
ICRP: International Commission on Radiological Protection
IQSC: Image quality scoring criteria
JND: Just noticeable difference
MTF: Modulation transfer function
NNPS: Normalized noise power spectrum
VP: Ventriculoperitoneal
