# Supplementary material for: Development and validation of image quality scoring criteria (IQSC) for pediatric CT: a preliminary study
Source: Insights Imaging. 2019 Sep 23;10:95. doi: 10.1186/s13244-019-0769-8 (PMC6757090; doi:10.1186/s13244-019-0769-8)
Supplement: Supplementary file 1 — Image quality scoring criteria (IQSC) for pediatric CT. (DOCX 23 kb) [file 13244_2019_769_MOESM1_ESM.docx]

**Additional file 1: Image Quality Scoring Criteria (IQSC) for Pediatric CT**

**Routine Chest CT**

Please do not include patients who have any metallic implants (prosthesis or splints): small surgical clips or sutures not causing any major artifacts are fine.

Please record the position of the arms of the child (Arms or hands by the side of body or above the head)

**Chest trauma**

Ability to see the following structures

Lung windows:

- 1. Segmental bronchi
  2. Segmental pulmonary arteries
  3. Ground glass opacities
  4. Consolidation or atelectasis
  5. Pneumothorax
  6. Motion artifacts (3= Absent; 2=Do not affect interpretation; 1= Do affect interpretation)

Mediastinal soft tissue windows:

1. Pleural effusion
2. Mediastinal vessels
3. Pericardial effusion
4. Adrenal glands
5. Lower pole of any side kidney

**Empyema, Lung abscess, lung nodule**

Lung windows:

1. Walls of segmental bronchi
2. Segmental pulmonary vessels
3. Pulmonary nodules (≥ 5mm)
4. Ground glass opacities
5. Consolidation
6. Motion artifacts (3= Absent; 2=Do not affect interpretation; 1= Do affect interpretation)

Mediastinal soft tissue windows:

1. Pleural effusion
2. Pleural thickening or enhancement (if it is post contrast chest CT)
3. Mediastinal lymph nodes (any size)
4. Adrenal glands
5. Lower pole of any side kidney

**Mediastinal vascular anomaly**

Lung windows:

1. Segmental bronchi
2. Trachea and mainstem bronchi
3. Segmental pulmonary arteries
4. Consolidation or atelectasis
5. Motion artifacts (3= Absent; 2=Do not affect interpretation; 1= Do affect interpretation)

Mediastinal soft tissue windows:

1. Pulmonary arteries (up to lobar level)
2. Pulmonary veins (near the drainage point in the left atrium or other veins)
3. Aorta and its main branches
4. Adrenal glands
5. Lower pole of any side kidney

**Mass evaluation**Lung windows:

1. Walls of segmental bronchi
2. Segmental pulmonary vessels
3. Pulmonary nodules (greater than or equal to 4 mm in size)
4. Ground glass opacities (greater than or equal to 4 mm in size)
5. Lung mass
6. Motion artifacts (3= Absent; 2=Do not affect interpretation; 1= Do affect interpretation)

Mediastinal soft tissue windows:

1. Pleural effusion
2. Mediastinal lymph nodes (any size)
3. Mediastinal vessels
4. Mediastinal mass
5. Adrenal glands
6. Lower pole of any side kidney

**Interstitial lung pathology**

Lung windows:

1. Walls of segmental bronchi
2. Segmental pulmonary vessels
3. Bronchiectasis
4. Ground glass opacities (greater than or equal to 4 mm in size)
5. Interlobular septal thickening
6. Motion artifacts (3= Absent; 2=Do not affect interpretation; 1= Do affect interpretation)

Mediastinal soft tissue windows:

1. Mediastinal lymph nodes (any size)
2. Pleural effusion
3. Adrenal glands
4. Lower pole of any side kidney

**Routine Abdomen CT**

Please do not include patients who have any metallic implants (prosthesis or splints): small surgical clips or sutures not causing any major artifacts are fine.

Please record the position of the arms of the child (Arms or hands by the side of body or above the head)

**Trauma or intrabdominal fluid collection not seen with ultrasonography**

1. Liver parenchyma
2. Portal veins
3. Common hepatic duct
4. Adrenal glands
5. Pancreatic duct
6. Ascites
7. Intraperitoneal free air

**Appendicitis/Volvulus /0bstruction**

1. Bowel wall
2. Liver parenchyma
3. Portal veins
4. Common hepatic duct
5. Adrenal glands
6. Pancreatic duct
7. Ascites
8. Intraperitoneal free air
9. Appendix

**Kidney Stone CT**

*Give one overall image quality score based on ability to see the following structures:*

1. Urinary tract calculi (location and size)
2. Atherosclerotic calcification in aorta or its branches
3. Upper ureters
4. Urinary bladder wall

*Please highlight reason for scores of 1 or 2: Poor image quality (for reasons others than from artifacts or poor contrast enhancement) Too much altered image appearance OR Motion artifacts OR Too much streak artifacts*

**Craniosynostosis**

1. Bone windows:
   1. Cortical and trabecular bone structures in the skull
   2. Cranial sutures
2. Brain window
   1. Lateral ventricle
   2. Third ventricle
   3. Fourth ventricle
   4. Extra-axial cerebrospinal (CSF) spaces
3. Motion artifacts (3= Absent; 2=Do not affect interpretation; 1=Affect interpretation)

**VP Shunt Patency/ Hydrocephalus**

1. Brain Windows:
   1. Lateral ventricles
   2. Third ventricle
   3. Fourth ventricle
   4. Extra-axial cerebrospinal (CSF) spaces
2. Motion artifacts (3= Absent; 2=Do not affect interpretation; 1=Affect interpretation)
